# Non-occupational lead poisoning associated with traditional Chinese medicine: A case report

**DOI:** 10.3389/fpubh.2022.938186

**Published:** 2022-09-13

**Authors:** Huan Ma, Li-meng Wu, Yu Zou, Xiao-an Li

**Affiliations:** ^1^Department of Gastroenterology, Mianyang Central Hospital, School of Medicine, University of Electronic Science and Technology of China, Mianyang, China; ^2^Department of Burn and Plastic Surgery, Mianyang Central Hospital, School of Medicine, University of Electronic Science and Technology of China, Mianyang, China

**Keywords:** periumbilical pain, anemia, lead poisoning, traditional Chinese medicine, case report

## Abstract

**Introduction:**

Traditional Chinese medicine has a long history and is widely popular in China because of its safety and small side effects. In Chinese families, people believe that the combination of traditional Chinese and Western medicine is more effective, and in terms of conditioning and health care, they tend to rely on traditional Chinese medicine. However, the toxic and side effects of traditional Chinese medicine, especially heavy metal poisoning, should not be ignored.

**Patient concerns:**

A case of non-occupational lead poisoning caused by long-term use of traditional Chinese medicine.

**Diagnosis:**

A 21-year-old man with severe colic periumbilical pain was referred to our hospital. Through careful inquiry of his medical history, we found that he had been taking traditional Chinese medicine to treat facial acne in the past year. His test results showed anemia, liver damage, blood lead concentration of 1,268.4 μg/L, and bone marrow smear showed basophilic stippling erythrocyte. The patient was diagnosed with “lead poisoning.”

**Interventions:**

The patient was given treatment with lead driving.

**Outcomes:**

The patient recovered after treatment.

**Conclusion:**

We found that lead poisoning in patients taking traditional Chinese medicine has been reported from time to time. Through consulting the data, we summarized the most common drugs leading to lead poisoning, and reviewed the pathogenesis and common clinical manifestations of lead poisoning. Because lead poisoning is easy to be misdiagnosed, we should ask more carefully about the past history and drug history of patients in order to make timely diagnosis and treatment.

## Introduction

Globally, Eastern medicine has become a popular alternative therapy for preventing or curing disease. The World Health Organization (WHO) reported the use of Eastern medicine by 50–90% of the population in some Asian countries in 2008 (1). Traditional Chinese medicine is an important part of Eastern medical science and culture. There are 12,807 kinds of traditional Chinese medicine resources in China. The market scale of Chinese herbal medicine in 2017 was US$ 14.76 billion yuan, which reached nearly US$ 18.07 billion yuan in 2018. It is estimated that it will reach US$ 24.77 billion yuan in 2022 and more than US$ 29 billion yuan in 2024. The popularity of traditional Chinese medicine has also aroused public concern. In recent years, there have been reports on the potential toxicity and side effects of traditional Chinese medicine, including the adulteration and the contamination of traditional Chinese medicine, liver and kidney function damage and heavy metal poisoning (2–5). We report here a case of lead poisoning caused by long-term use of traditional Chinese medicine to remind the public that the safety of traditional Chinese medicine should not be ignored.

## Case report

**Figure 1:**
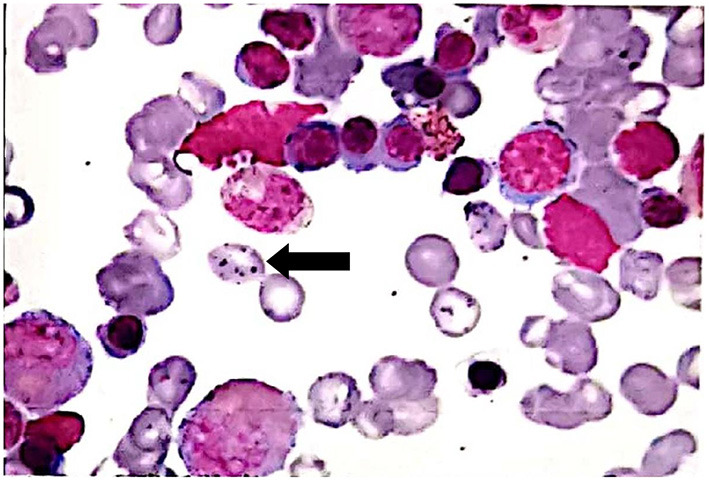
The bone marrow smear showed basophilic stippling erythrocyte (arrow).

A 21-year-old man with severe colic periumbilical pain was referred to our hospital. He complained of paroxysmal periumbilical colic with constipation for 20 days. Two weeks before he came to our hospital, he went to another local hospital, where a colonoscopy and an abdominal ultrasound were performed and nothing abnormal was observed. The abdominal X-ray indicated irritable bowel syndrome. He was diagnosed with incomplete intestinal obstruction and was discharged after symptomatic treatment. However the symptoms persisted, so the patient was referred to our hospital. On physical examination, the patient's vital signs, neurological examination and chest examination were normal. The abdominal examination revealed hypoactive bowel sounds and mild diffuse abdominal tenderness without rebound. Laboratory tests revealed moderate normocytic anemia (hemoglobin concentration of 89 g/L). Ferritin was moderately elevated, but the folate and vitamin B12 levels were normal. The Coombs test was negative. A liver function test showed hyperbilirubinemia (total bilirubin level of 43.6 μmol/L, indirect bilirubin level of 27.1 μmol/L) and a slightly increased level of liver transaminases (alanine aminotransferase level of 51 U/L, aspartateaminotransferase level of 57 U/L, gamma glutamyl transpeptidase level of 164 U/L, alkaline phosphatase level of 143 U/L). Tests for autoimmune diseases showed negative results. The urine and stool tests yielded normal results. An abdominal computerized tomography (CT) showed right inferior mesenteric lymph nodes. Sacral 1 occult spina bifida was found on the scan. A gastroscopy showed polyps of the fundus gland and chronic non-atrophic gastritis. After the treatment of spasmolysis and defecation, the patient's symptoms were not relieved. We noticed that the patient's main clinical manifestations were abdominal pain, constipation and hemolytic anemia, so we asked the patient again about his past and recent medication history. We found that the patient was treated with oral Chinese medicine powder and Chinese medicine pills for facial acne in the last year. Therefore, we thought the diagnosis could be lead poisoning, so we sent for a blood lead test. The results revealed an elevated blood lead level of 1,268.4 μg/L (normal level <400 μg/L). To confirm the typical clinical manifestations of lead poisoning, we performed a bone marrow smear, and the results showed that there were many basophilic stippling erythrocytes (Figure 1). The patient was diagnosed with lead poisoning. The patient immediately stopped taking traditional Chinese medicine and received lead chelating therapy. His periumbilical pain and constipation were noticeably relieved after treatment and his blood lead level returned to normal after two rounds of treatment. We detected the concentration of lead in the traditional Chinese medicine taken by the patient, and found that the concentration of lead in the pills was 8,421 μg/g, which is a high concentration reported in the cases of lead poisoning caused by the traditional Chinese medicine published in China and abroad.

## Discussion

The main components of traditional Chinese medicine come from plants, animals, minerals and other substances in nature, which are commonly known as “natural drugs.” Before human society learned how to cultivate these substances, they were used as food. Therefore, traditional Chinese medicine is also known to be “safe and has fewer side effects.” In Chinese families, people believe that the combination of traditional Chinese and Western medicine is more effective, and in terms of conditioning and health care, they tend to rely on traditional Chinese medicine, which makes Chinese medicine widely popular in China. In this year's new coronavirus epidemic prevention and control work, Chinese medicine has also played a pivotal role, which shows the trust and favor of traditional Chinese medicine by Chinese people. In addition, the popularity of Chinese herbal medicine in South Korea, India and other places is similar to that in China, and with the persence of Chinese people worldwide, the popularity of traditional Chinese medicine is promoted around the world. At the same time, the popularity of traditional Chinese medicine also causes the public to pay attention to its safety. In recent years, in addition to the toxicity of drugs, excessive dosage, incompatibility and other aspects, heavy metal poisoning caused by traditional Chinese medicine has also been reported. Common heavy metal poisoning mainly includes arsenic, lead, and mercury. After consulting the data, we found that lead poisoning caused by traditional Chinese medicine is more common in children. It is mainly oral administration of a kind of traditional Chinese medicine called “jineijin,” which is mainly used for food retention and plays a role in helping digestion. Testing has shown there is a high concentration of lead in “jineijin.” Another use of traditional Chinese medicine is the external use of “Hongdan,” which is mainly used to treat eczema and plays a role in skin care. Hongdan is the official term for red lead (Pb3O4) according to the pharmacopeia of traditional Chinese medicine (5).

**Figure 2:**
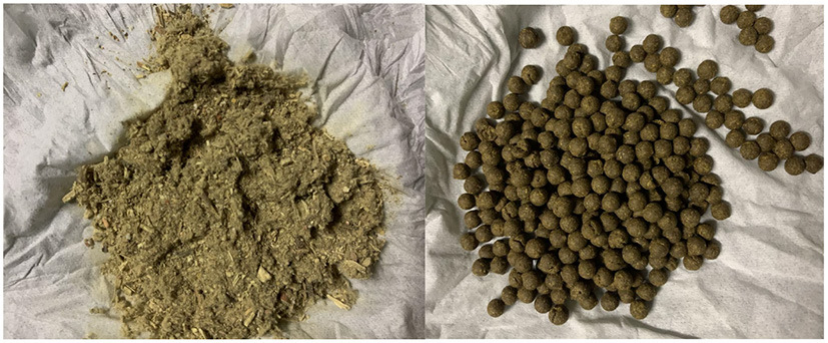
The patient took traditional Chinese medicine powder and pills.

In this case, traditional Chinese medicine was mainly used to treat facial acne. The patient used powder and pills (Figure 2). We detected that the lead content in the powder was 5.85 μg/g, while the lead content in the pills was 8,421 μg/g. The patient had taken approximately 20 grams of pills and 100 grams of powder each week. According to the Joint Food and Agriculture Organization and the WHO Expert Committee on Food Additives in 1986, provisional tolerable weekly Intake for lead from all sources is 25 μg per kilogram of body weight (6). The patient weighed 60 kg and could tolerate 1,500 μg of lead a week. Therefore, lead intake from TCM in this case was hundreds of times more than the recommended amount.

After the long-term oral administration of a large dose of lead, the blood lead level that accumulates in the body gradually increases, leading to damage in the digestive, circulatory, nervous and other systems.

Lead is an electropositive metal. Its high affinity for negatively charged sulfhydryl groups leads to the inhibition of sulfhydryl-dependent enzymes, such as γ-aminolevulinic acid dehydrogenase and ferrochelatase in heme synthesis. This disruption of hemoglobin synthesis leads to the production of free erythrocyte protoporphyrins that can be measured (7). Therefore, some lead poisoning may be misdiagnosed as acute porphyria. In addition, the inhibition of pyrimidine 5'-nucleotidase by lead can cause the degradation of ribosomal RNA in red blood cells, which can cause basophilic stippling shown on a peripheral smear and bone marrow smear. Basophilic stippling was an important clue to the diagnosis of lead poisoning in this case. Basophilic stippling in a bone marrow smear suggests lead intoxication, but is non-specific.

Divalent lead ions can also compete with calcium ions in some biological systems, such as mitochondrial respiration and multiple neural functions. It has been found that lead interferes with several calcium dependent processes and activates PKC, which is the promoting mechanism of lead neurotoxicity (7). Lead changes the permeability of blood-brain barrier and accumulates in astrocytes, which are essential for maintaining neuronal environment (8). Lead can also affect DNA and RNA, but the mechanism is not fully understood. Some studies have shown that lead exposure may change the overall DNA methylation level (9, 10). Lead has an effect on cell membrane and interferes with many energy systems and transport systems, which may be the reason for the effect of shortening the lifespan of red blood cells, hemolysis and nephrotoxicity (7).

Lead poisoning may produce a series of clinical manifestations, including fatigue, abdominal pain, headache, nausea, constipation, anemia, irritability, subtle mood changes, and pain in the hands, feet, muscles, or joints (11). These symptoms are not specific, and physical examinations show no warning signs in patients with lead poisoning.

Most of the cases of lead poisoning are occupational diseases, which are closely related to the working environment and contact time of the patients. Moreover, the patients with occupational sensitivity and deep understanding of lead poisoning are often easy to diagnose and receive timely treatment. The incidence rate of non-occupation lead poisoning caused by Chinese medicine preparation is low, but it is reported in clinical practice. Because the patients have mostly non-specific symptoms such as abdominal pain and constipation, and they may not provide the history of poisoning voluntarily, they are easily misdiagnosed and missed in the clinical setting.

Heavy metal poisoning caused by traditional Chinese medicine often comes from inferior medicinal materials or pollution in the production process (1, 5). In the end, we traced the source of traditional Chinese medicine in this case, found that the excessive metal lead in the traditional Chinese medicine was polluted during the preparation of traditional Chinese medicine powder and pill. Therefore, the preparation of the traditional Chinese medicine was also stopped.

## Conclusion

Traditional Chinese medicine plays an important role in the treatment and prevention of diseases, but when a patient is taking Chinese medicine, we should be alerted to the side effects of the drugs. The main manifestations of non-occupational lead poisoning caused by traditional Chinese medicine are abdominal pain, constipation, anemia and other non-specific symptoms, which are easily misdiagnosed and missed in the clinical setting. Therefore, we should ask more carefully about past histories and drug histories of patients, so as to diagnose and provide treatment in time and avoid further injury to the patients.

In addition to lead, arsenic, mercury and other heavy metals related to traditional Chinese medicine also cause great harm to human body. Therefore, we recommend that doctors pay attention to the dosage of drugs when prescribing, and that patients be alert to the possibility of heavy metal poisoning when taking drugs, and urge the public to pay more attention to the safety of traditional Chinese medicine.

## Data availability statement

The raw data supporting the conclusions of this article will be made available by the authors, without undue reservation.

## Ethics statement

The studies involving human participants were reviewed and approved by Ethics Committee of Mianyang Central Hospital. The patients/participants provided their written informed consent to participate in this study.

## Author contributions

HM: conceptualization, data curation, and writing—original draft. L-mW: data curation, visualization, and investigation. YZ: methodology, investigation, and writing—original draft. X-aL: supervision and writing—review and editing. All authors contributed to the article and approved the submitted version.

## Conflict of interest

The authors declare that the research was conducted in the absence of any commercial or financial relationships that could be construed as a potential conflict of interest.

## Publisher's note

All claims expressed in this article are solely those of the authors and do not necessarily represent those of their affiliated organizations, or those of the publisher, the editors and the reviewers. Any product that may be evaluated in this article, or claim that may be made by its manufacturer, is not guaranteed or endorsed by the publisher.

## Abbreviations

WHO: World Health Organization
CT: computerized tomography
TCM: Traditional Chinese medicine
RNA: ribose nucleic acid
PKC: protein kinase C
DNA: deoxyribonucleic acid.
